# Not All Lectins Are Equally Suitable for Labeling Rodent Vasculature

**DOI:** 10.3390/ijms222111554

**Published:** 2021-10-26

**Authors:** Roberta Battistella, Marios Kritsilis, Hana Matuskova, Douglas Haswell, Anne Xiaoan Cheng, Anja Meissner, Maiken Nedergaard, Iben Lundgaard

**Affiliations:** 1 Department of Experimental Medical Science, Faculty of Medicine, Lund University, 22362 Lund, Sweden; roberta.battistella@med.lu.se (R.B.); marios.kritsilis@med.lu.se (M.K.); hana.matuskova@med.lu.se (H.M.); anja.meissner@med.lu.se (A.M.); 2 WCMM Wallenberg Centre for Molecular Medicine, Faculty of Medicine, Lund University, 22362 Lund, Sweden; 3 German Center for Neurodegenerative Diseases, 53127 Bonn, Germany; 4 Department of Neurology, Division of Vascular Neurology, University Hospital Bonn, 53127 Bonn, Germany; 5 Center for Translational Neuromedicine, Department of Neurosurgery, University of Rochester Medical Center, Rochester, NY 14642, USA; douglashaswell@gmail.com (D.H.); anne.cheng@westernu.edu (A.X.C.); Maiken_Nedergaard@urmc.rochester.edu (M.N.); 6 Center for Basic and Translational Neuroscience, Faculty of Health and Medical Sciences, Neurology Department, University of Copenhagen, 2200 Copenhagen, Denmark

**Keywords:** lectins, lectin angiography, blood vessels, vascular research, stroke, angiogenesis

## Abstract

The vascular system is vital for all tissues and the interest in its visualization spans many fields. A number of different plant-derived lectins are used for detection of vasculature; however, studies performing direct comparison of the labeling efficacy of different lectins and techniques are lacking. In this study, we compared the labeling efficacy of three lectins: *Griffonia simplicifolia* isolectin B4 (IB4); *wheat germ agglutinin* (WGA), and *Lycopersicon esculentum agglutinin* (LEA). The LEA lectin was identified as being far superior to the IB4 and WGA lectins in histological labeling of blood vessels in brain sections. A similar signal-to-noise ratio was achieved with high concentrations of the WGA lectin injected during intracardial perfusion. Lectins were also suitable for labeling vasculature in other tissues, including spinal cord, dura mater, heart, skeletal muscle, kidney, and liver tissues. In uninjured tissues, the LEA lectin was as accurate as the Tie2–eGFP reporter mice and GLUT-1 immunohistochemistry for labeling the cerebral vasculature, validating its specificity and sensitivity. However, in pathological situations, e.g., in stroke, the sensitivity of the LEA lectin decreases dramatically, limiting its applicability in such studies. This work can be used for selecting the type of lectin and labeling method for various tissues.

## 1. Introduction

The first accurate description of the cardiovascular system dates back to 1628 when the pioneering work of William Harvey revolutionized Galen’s theory by centering the movement of blood around the heart as a central pump that pushes blood through a network of conduit vessels, nourishing tissues and organs [1]. In 1661, Marcello Malpighi discovered capillaries as connections between arteries and veins, revising Harvey’s model of blood circulation. Since then, many discoveries have improved the complex understanding of the cardiovascular system.

The early stages of cardiovascular development begin in the embryonic mesoderm when angiocysts start forming a primitive endothelial heart tube, preceding the formation of blood vessels through substantial embryonic angiogenesis, followed by pruning and remodeling of the vessels. Although the vasculature develops as one of the first organ systems, reorganization of the vascular system still occurs in adults during different physiological and pathological situations, such as the ovarian cycle and the formation of tumors [2]. Furthermore, in the brain, blood vessel angiogenesis and remodeling occur in adult life, for instance, as a response to augmented vascular endothelial growth factor (VEGF) and hypoxia-inducible factor 1 (HIF-1) signaling [3]. Particularly, in response to chronic hypoxia, compensatory vascular mechanisms are activated, leading to an increase in the number, elongation, and enlargement of capillaries [4]. Angiogenesis, and neovascularization constitute a common response to tissue injury, characteristic of several neurological diseases including stroke [5], traumatic brain injury [6], and Parkinson’s disease (PD) [7,8]. In the aged and vulnerable brain, angiogenesis mechanisms seem to fail [9] and often mark an important risk factor for developing cerebrovascular diseases associated with white matter damage in humans [10]. The vasculature is capable of dynamic adaptation, and this characteristic is of importance to all body tissues. Therefore, it is essential for researchers to understand and investigate the integrity of the vascular system’s structure and functionality.

To visualize blood vessels, immunohistochemical techniques targeting endothelial markers such as cluster of differentiation (CD) 31 [11,12,13] or basement membrane markers such as laminin [14,15] have been widely used. More recently, methods including intravenous injection of fluorescent dyes followed by in vivo two-photon imaging [16,17,18,19] or intravenous injection of polymerizing contrast agents followed by ex vivo microCT [20] have been developed.

Interestingly, lectins have been used to label vasculature through immunohistochemical techniques [21,22,23], as well as direct intravascular delivery [15,24,25,26]. Indeed, the use of plant-derived lectin proteins for the detection of vasculature dates back to 1982, when it was reported that lectins bind to the luminal part of the capillary endothelium in the mouse pancreas and intestinal mucosa through interactions with sugar residues [27]. As lectins are glycoproteins with a carbohydrate recognition domain, it is not surprising that pretreatment with the specific competitive monosaccharides alone was able to impede lectin binding. Shortly after, lectins were used to label mouse cerebral blood vessels through direct injection in the blood circulation via intracardial perfusion [28], and this approach nowadays is the basis for more sophisticated techniques, including the imaging of vasculature using intravascular lectin perfusion followed by optical clearing and light sheet microscopy [29]. Various lectins have been used as an alternative to the commonly used markers for staining blood vessels in different tissues [15,21,24,26,30,31]. However, studies performing direct comparison of the labeling efficacy of different lectins and their different staining methods as well as studies comparing lectins’ specificity towards blood vessels in physiological and pathological conditions are lacking, thus hampering the comparison of results between different studies. 

Here, we conducted a comprehensive analysis of the labeling efficacy of three of the most commonly used lectins (*Griffonia simplicifolia* isolectin B4 (IB4); *wheat germ agglutinin* (WGA); *Lycopersicon esculentum agglutinin* (LEA)), and compared the techniques, histological application vs. intracardial perfusion, for labeling blood vessels in different tissues with a focus on the cerebral vasculature. We also applied lectin labeling to a stroke model.

## 2. Results

### 2.1. Histological Staining of Cerebral Blood Vessels with the LEA Lectin Gives a Better Signal-to-Noise Ratio than WGA and IB4 Lectins

The IB4, WGA, and LEA lectins have all been used to either label vasculature upon binding to endothelial cells following intravascular delivery [24,25,26,30,32] or for labeling blood vessels using immunohistological techniques [15,21,22,23,33].

**Figure 1:**
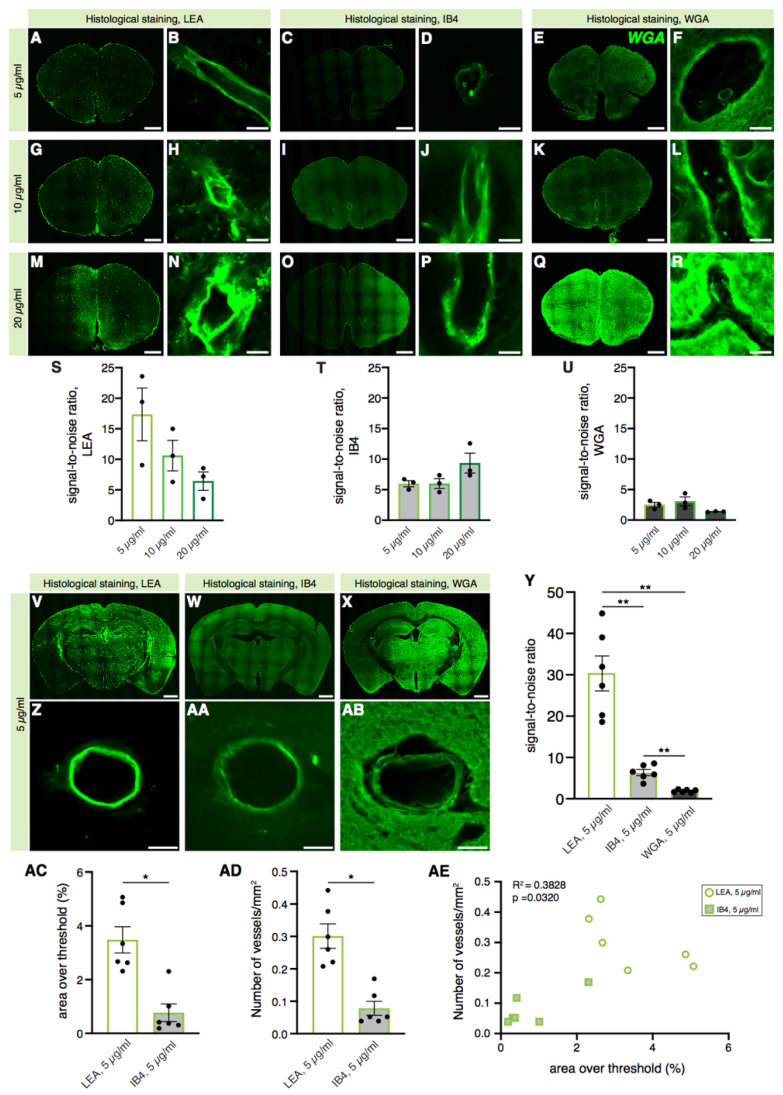
Labeling of blood vessels in free-floating brain sections by lectins *Lycopersicon esculentum agglutinin* (LEA), *Griffonia simplicifolia* isolectin B4 (IB4), and *wheat germ agglutinin* (WGA). (**A**–**R**) Representative confocal images of mouse brain sections histologically stained with the LEA, IB4, and WGA lectins at different concentrations. (**S**–**U**) Quantification of the signal-to-noise ratio. Each datapoint represents a single vessel; *n* = 1. (**V**–**AB**) Representative confocal images of (i) 10× mouse brain sections histologically stained with the LEA, IB4, and WGA lectin solutions at a concentration of 5 µg/mL (**V**,**W**,**X**) and (ii) high magnification (20×) of the blood vessels (**Z**,**AA**,**AB**) used for the quantification of the signal-to-noise ratio in (**Y**). Three vessels analyzed per animal, *n* = 6 mice (repeated measures one-way ANOVA, *p* = 0.0010; Tukey’s multiple comparisons test: WGA vs. IB4, *p* = 0.0039; WGA vs. LEA, *p* = 0.0025; IB4 vs. LEA, *p* = 0.0034). ** = *p* < 0.01. (**AC**–**AD**) Quantification of the area over the threshold (%) (**AC**) and of the number of vessels/mm^2^ (**AD**) labeled by the IB4 and LEA lectins in brain sections. Each datapoint represents the averaged values in one single animal; *n* = 6 mice. (AC: Wilcoxon test, *p* = 0.0312; AD: Wilcoxon test, *p* = 0.0312). * = *p* < 0.05. (**AE**) Graph showing the existing correlation between the percentage of the area over the threshold and the number of vessels/mm^2^ labeled with the IB4 and LEA lectins in brain sections. Each datapoint represents the averaged values in one single animal; *n* = 6 mice (correlation, Pearson coefficient: R^2^ = 0.3828, *p* = 0.0320). Scale bars: 1000 µm for low-magnification images, 10 µm for high-magnification images.

Here, we compared the specificity of histological labeling of the mouse vasculature of the most commonly used lectins, WGA, IB4, and LEA (Figure 1). The labeling efficacy was calculated as the ratio of the averaged peak signal at the vascular walls to the background signal (signal-to-noise ratio) of single vessels. In order to determine the lectin concentration with the most appropriate signal-to-noise ratio, we tested different lectin concentrations (5 µg/mL; 10 µg/mL; 20 µg/mL) for WGA, IB4, and LEA staining using 100 µm free-floating mouse brain sections (Figure 1A–R).

The lowest lectin concentration (5 µg/mL) revealed the lowest background labeling in all lectin stainings (Figure 1S–U) and thus was chosen for the comparison of the LEA, IB4, and WGA signal-to-noise ratios in mouse brain vessels in confocal images at 20× magnification (Figure 1V–AB). Our results (Figure 1Y) demonstrate that the LEA lectin showed the best efficacy in labeling blood vessels in mouse brain sections and is by far superior to the other commonly used lectins for this type of applications (Figure 1Y; WGA = 1.882 ± 0.1366; IB4 = 6.363 ± 0.7391; LEA = 30.34 ± 4.238). Moreover, the analysis of the LEA’s signal-to-noise ratio in labeling longitudinally vs. coronally sectioned vessels (Figure S1A–C) showed a nonsignificant tendency towards higher values in the longitudinally sectioned vessels (signal-to-noise ratio in coronally sectioned vessels: 8.525 ± 0.6112; in longitudinally sectioned vessels: 13.66 ± 1.758). On the other hand, the WGA lectin histological staining showed diffuse and unspecific labeling of vascular walls and the parenchyma, resulting in a very high background and a lower signal-to-noise ratio. Compared to the LEA lectin staining, the IB4 lectin covered a lower percentage of the area (Figure 1AC, IB4 = 0.7673 ± 0.3292; LEA = 3.484 ± 0.4896) and labeled overall a lower number of blood vessels (Figure 1AD, IB4 = 78.12 ± 21.79; LEA = 301.6 ± 37.65), elevating the LEA lectin’s value as a useful tool for labeling cerebral blood vessels. When plotted together, the percentage of the area covered by the staining and the number of blood vessels counted per mm^2^ showed not only a positive correlation (Figure 1AD, R^2^ = 0.3828, *p* = 0.0320), but also the presence of two distinct clusters representing IB4 and LEA staining. We also confirmed that autofluorescence did not alter the LEA lectin’s signal-to-noise ratio in comparison with the signal from a tissue that was not labeled with the lectin (Figure S1D–F). Using the same confocal settings (Figure S1D), we could not detect any signal in the 488 spectrum, and only by increasing the settings ten times we were able to detect tissue autofluorescence (Figure S1E).

Taken together, these results indicate that the LEA lectin is preferable over the IB4 and WGA lectins when used to label blood vessels in free-floating brain sections with histological approaches.

### 2.2. A Higher Signal-to-Noise Ratio Can Be Achieved by Intracardiac Perfusion of Lectins

**Figure 2:**
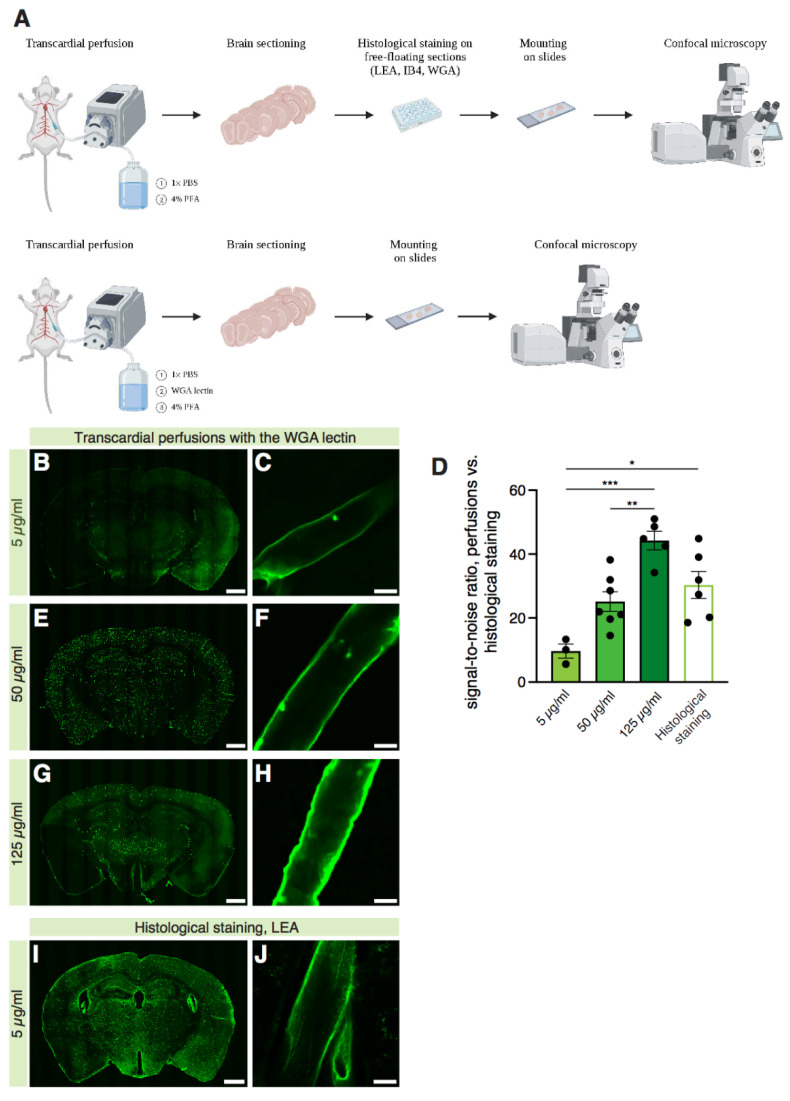
Comparison of different methods to labeling brain vessels with lectins: intravascular WGA delivery during terminal perfusion vs. LEA histological staining on free-floating sections. (**A**) Schematic drawing summarizing the setup for the experiments comparing the two methods. Created with BioRender. (**B**–**J**) Representative 10× confocal images of brain sections of the mice intracardially perfused with the WGA lectin at a concentration of 5 µg/mL (**B**), 50 µg/mL (**E**), and 125 µg/mL (**G**) compared to the ones histologically stained with the LEA lectin (**I**) and (**D**) quantification of the signal-to-noise ratio of the vessels stained with different methods (representative confocal 20×-magnified images in **C**,**F**,**H**,**J**). Three vessels analyzed per animal; *n* = 3 mice for the 5 µg/mL group, *n* = 7 mice for the 50 µg/mL group, *n* = 5 mice for the 125 µg/mL group, *n* = 6 mice for the LEA histological staining group (one-way ANOVA test, *p* = 0.0002; Tukey’s multiple comparisons test: 5 µg/mL WGA vs. 125 µg/mL WGA, *p* = 0.0001; 5 µg/mL WGA vs. LEA, *p* = 0.0112; 50 µg/mL WGA vs. 125 µg/mL WGA, *p* = 0.0047). * = *p* < 0.05; ** = *p* < 0.01; *** = *p* < 0.001. Scale bars: 1000 µm for low-magnification images, 10 µm for high-magnification images.

With a molecular weight of 35 kDa, the WGA lectin does not cross the blood–brain barrier and can therefore be used to label the cerebral vasculature when administered intravascularly during terminal transcardial perfusion [29], eliminating parenchymal binding. Its relative cost efficiency compared to other lectins, e.g., IB4 and LEA, makes WGA a suitable candidate for methods that require a high volume of the lectin solution, i.e., a direct injection in the mouse bloodstream during terminal transcardial perfusion. We investigated the efficacy of WGA-mediated blood vessel labeling at different concentrations when intracardially perfused and compared it to the histological staining with the LEA lectin on free-floating brain sections (Figure 2A).

Different concentrations of the WGA lectin solution (Figure 2B–H: 5 µg/mL; 50 µg/mL; 125 µg/mL) were infused in the different groups of mice, and the signal-to-noise ratio of single vessels was assessed. The quantification showed that the WGA lectin had a concentration-dependent efficacy of staining blood vessels when intracardially perfused, with the best signal-to-noise ratio value in the 125 µg/mL group (Figure 2D, 5 µg/mL WGA = 9.659 ± 2.226; 50 µg/mL WGA = 25.16 ± 3.074; 125 µg/mL WGA = 44.25 ± 2.906).

In order to assess whether perfusion or histological staining would be preferable to achieve the best signal-to-noise ratio, a comparison was made between the two methods. The LEA lectin (Figure 2I–J: 5 µg/mL) was chosen for the histological staining method since it resulted in the best labeling efficacy on free-floating brain sections in our experimental setup compared to the IB4 and WGA lectins, respectively (Figure 1). The quantification (Figure 2D) showed that intracardiac perfusion of the WGA lectin at high concentrations (i.e., 50 and 125 µg/mL WGA) had a similar signal-to-noise labeling efficacy as the LEA lectin in the histological labeling method (50 µg/mL WGA = 25.16 ± 3.074; 125 µg/mL WGA = 44.25 ± 2.906; LEA = 30.34 ± 4.238). These results indicate that although WGA showed the lowest specificity for cerebral blood vessels when applied directly on free-floating brain sections, it can be used for specific labeling of the brain vasculature when applied intravascularly at high concentrations. However, when intracardially injected at low concentrations (i.e., 5 µg/mL), the WGA lectin shows a significant lower signal-to-noise ratio compared to the LEA lectin’s histological staining (5 µg/mL WGA = 9.659 ± 2.226; LEA = 30.34 ± 4.238). In conclusion, several methods for lectin-based labeling of blood vessels are applicable with similar efficacy, enabling the choice of the most suitable method for the experimental design of each individual study.

### 2.3. Lectin Is Widely Applicable for Labeling Blood Vessels in Various Tissues

**Figure 3:**
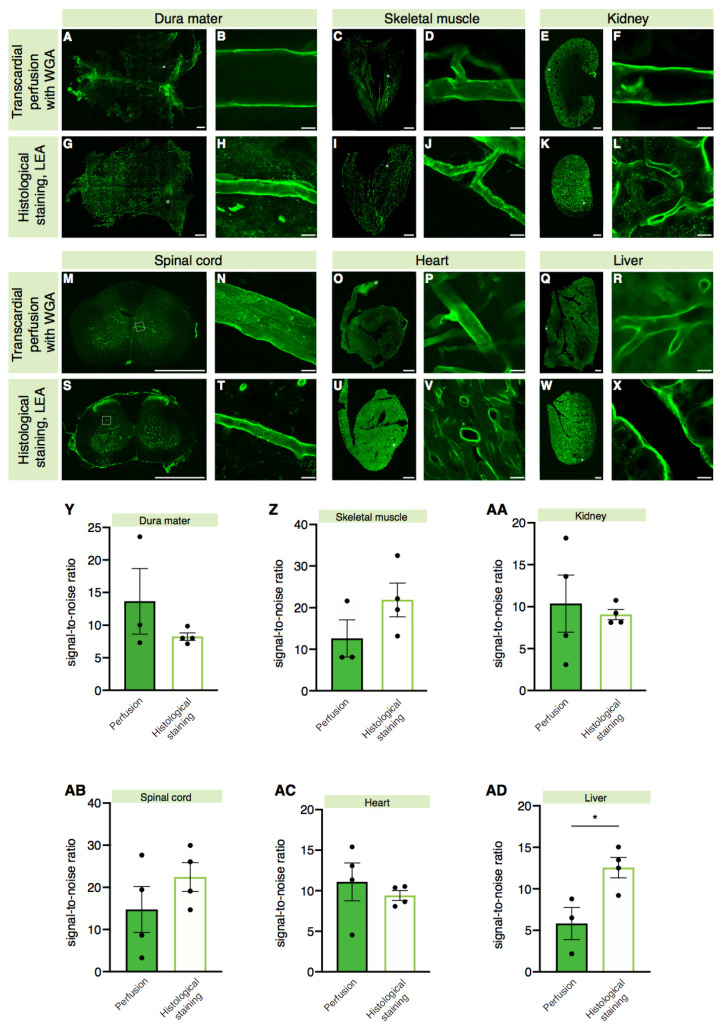
Comparison of different methods of labeling blood vessels in various organs with lectins: WGA intravascular delivery during terminal perfusion vs. LEA histological staining on free-floating sections. (**A**–**X**) Representative images of the whole-mount dura mater meninx, skeletal muscle, kidney, spinal cord, heart, and liver tissues of the mice intracardially perfused with the WGA lectin (concentration: 50 µg/mL) compared with those histologically stained with the LEA lectin. White squares indicate the point where the high magnification images of the blood vessels used for the quantification were acquired from. Scale bars: 1000 µm for low magnification images, 10 µm for high magnification images. (**Y**–**AD**) Quantification of the signal-to-noise ratio values of the two methods in different organs (dura mater: Welch’s *t*-test, *p* = 0.3955; skeletal muscle: Mann–Whitney U test, *p* = 0.2286; Kidney: Welch’s *t*-test, *p* = 0.7319; spinal cord: unpaired *t*-test, *p* = 0.2778; heart: unpaired *t*-test, *p* = 0.5096; liver: unpaired *t*-test, *p* = 0.0272); *n* = 3 to 4 mice for the 50 µg/mL WGA perfusion group and *n* = 4 mice for the LEA histological staining group. * = *p* < 0.05.

Next, we tested whether the labeling results obtained with lectins for the cerebral vasculature also applied to other organs. To assess a broad selection of tissues, we investigated labeling of the mouse dura mater, skeletal muscle, kidney, spinal cord, heart, and liver tissues (Figure 3A–X).

The analysis was performed comparing the lectin staining of the organs of two independent groups of mice. One group was perfused with 1× PBS followed by 4% PFA, and the whole-mount dura mater as well as tissue sections of the abovementioned organs were stained with the LEA lectin (5 µg/mL). The second group was intracardially perfused with the WGA lectin (50 µg/mL) to allow labeling of the blood vessels through direct binding of the WGA lectin to the luminal part of the endothelial cells before the organs of interest were harvested.

The labeling efficacy of histological staining with the LEA lectin and intracardial WGA (50 µg/mL) lectin perfusion was comparable in blood vessels of the majority of the organs analyzed, i.e., the dura mater, skeletal muscle, kidney, spinal cord, and heart (Figure 3Y–AC, dura mater: LEA = 8.259 ± 0.5733, 50 µg/mL WGA = 13.65 ± 5.021; skeletal muscle: LEA = 21.85 ± 4.023, 50 µg/mL WGA = 12.60 ± 4.497; kidney: LEA = 9.067 ± 0.6144, 50 µg/mL WGA = 10.36 ± 3.398; spinal cord: LEA = 22.45 ± 3.428, 50 µg/mL WGA = 14.76 ± 5.460; heart: LEA = 9.404 ± 0.6159, 50 µg/mL WGA = 11.10 ± 2.336). Thus, both staining methods can be used for the same purpose. In the liver, on the other hand, the LEA lectin staining showed a higher signal-to-noise ratio when compared to the WGA lectin-perfused sections (Figure 3AD, LEA = 12.56 ± 1.231, 50 µg/mL WGA = 5.823 ± 1.939). This is most likely due to the unique anatomy of blood vessels in the liver defined as “fenestrated” owing to the presence of open pores in the liver sinusoidal endothelial cells [34] which may cause leakage of the WGA lectin solution injected in the blood stream into the liver parenchyma. In the kidney, although the signal-to-noise ratio did not differ between the two labeling methods (Figure 3AA), LEA lectin histological staining (Figure 3L) revealed the characteristic glomeruli structures [35], which were not detectable in kidney sections obtained from the mice intracardially injected with the WGA lectin (Figure 3F), suggesting that the LEA lectin is not filtered in the glomeruli.

Interestingly, we detected that both the sagittal and the transverse sinuses in the dura mater were stained with the LEA lectin after histological staining of the tissue (Figure S2B), as well as with the intracardially injected WGA lectin (Figure S2C), showing that lectins label veins efficiently. Lectins can also efficiently label arteries, as shown by the colocalization of the LEA lectin’s signal with the alpha smooth muscle actin (α-SMA)-positive vessels (Figure S2D–E).

To assess whether lectins could label lymphatic vessels as well, we used the LEA lectin conjugated to AlexaFluor-647 (Figure S2F) to stain the whole dura of Prox1–eGFP reporter mice [36]. Using confocal imaging and 3D reconstructions thereof, we did not detect colocalization between the lectin and the Prox1–eGFP signal (Figure S2G–J; Supplementary Movie 1), suggesting that lectins do not label lymphatic vessels. 

In light of our results, albeit seemingly universally applicable, the unique characteristics of the organ of interest used for lectin-based labeling of blood vessels should be considered.

### 2.4. Lectin Labeling of Cerebral Blood Vessels Is Equivalent to Reporter Mice and Immunostaining

**Figure 4:**
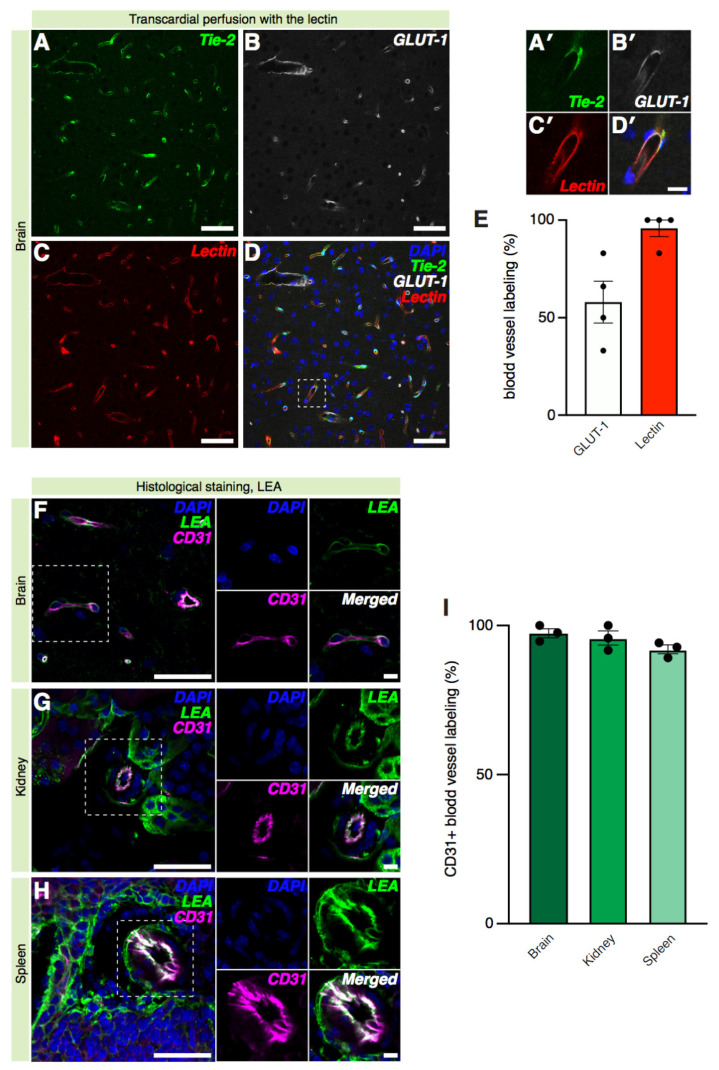
Comparison of lectin labeling of blood vessels in different types of the endothelium with the commonly used blood vessel markers. (**A**–**D**) Representative 20× confocal images of a brain section from a Tie2–eGFP reporter mouse intracardially perfused with the biotinylated lectin and processed with Cy3-conjugated streptavidin and GLUT-1 antibodies. Scale bars: 50 µm. The white dashed square marks the magnified inset in (**A**′–**D**′). Scale bars: 10 µm. (**E**) Quantification of blood vessels labeled (%) by GLUT-1 and lectin staining; *n* = 4 mice (Wilcoxon test, *p* = 0.2500). (**F**–**H**) Representative 20× confocal images of the brain, kidney, and spleen sections labeled with the CD31 endothelial marker. Scale bars: 50 µm. White dashed squares mark the magnified insets on the right. Scale bars: 10 µm. (**I**) Quantification of CD31^+^ blood vessels (%) in the brain, kidney, and spleen tissues that are co-labeled with LEA; *n* = 3 mice (one-Way ANOVA, *p* = 0.1894).

We then performed a more detailed investigation of the specificity of lectin labeling of blood vessels by comparing it to immunostaining against glucose transporter-1 (GLUT-1), a protein expressed at the blood–brain barrier site by endothelial cells and astrocytes. GLUT-1 is widely used to label the vasculature in the brain [37]. We also used Tie2–eGFP reporter mice, which carry an endogenous marker of endothelial cells [38]. The Tie2–eGFP reporter mice [39] were intravenously injected with biotinylated lectin (125 µg/mL) and perfused thereafter. Brain sections were processed with Cy3-conjugated streptavidin and the GLUT-1 antibody (Figure 4A–D′). 

In each section, blood vessels were identified by the presence of GLUT-1 staining and Tie2–eGFP expression. The presence of lectin co-staining was then quantified for the identified vessels. The application of Cy3-conjugated streptavidin to brain tissue sections from the mice intracardially perfused with biotinylated lectin revealed an extensive pattern of arterioles and capillaries (Figure 4C). The percentage of LEA-stained blood vessels was comparable to that of GLUT-1 labeled vessels (Figure 4E, lectin = 95.75 ± 4.250; GLUT-1 = 58.00 ± 10.72). Our results show that lectin labeling of blood vessels overlaps with GLUT-1 staining in the mouse brain and that the LEA lectin provides reliable labeling of blood vessels even when applied directly to the sectioned tissue. Next, we tested whether lectin sensitivity towards blood vessels could be altered in tissues characterized by endothelia with different characteristics than the brain. Therefore, we tested co-localization of another commonly used marker for endothelial cells, CD31, with LEA in different tissues: the brain as an example of continuous endothelium; the kidney as an example of fenestrated endothelium; and the spleen as an example of discontinuous endothelium. We identified blood vessels in the abovementioned tissues by positivity to CD31, and then we assessed the presence of LEA labeling on the same vessels (Figure 4F–H). Our results showed that 97.43 ± 1.541% of the CD31+ vessels were positive for lectin staining in the brain, while the results were 95.83 ± 2.406% for the kidney and 92.03 ± 1.489% in the spleen, suggesting that lectin sensitivity towards blood vessels does not depend on the intrinsic characteristics of the blood vessels’ endothelium (Figure 4I). Moreover, our results showed that while the specificity of the LEA lectin labeling of blood vessels in the brain is 94.62 ± 1.614, it decreases considerably in the kidney down to 58.48 ± 11.19 as the LEA lectin also labels the glomeruli in this organ (see also Figure 3L). The LEA lectin’s specificity was barely assessable in the spleen where the LEA lectin appears to label not only blood vessels, but also the structural reticular meshwork of the spleen, as previously shown to happen in the rat spleen with other lectins [40].

Therefore, lectin labeling of blood vessels in the brain is as reliable as labeling with commonly used endothelial cell markers. However, the intrinsic structure of some organs, e.g., of the kidney or the spleen, may represent a limitation to the ability of lectin to label specifically blood vessels.

### 2.5. LEA Lectin’s Specificity for Blood Vessel Labeling Decreases in the Ischemic Brain

**Figure 5:**
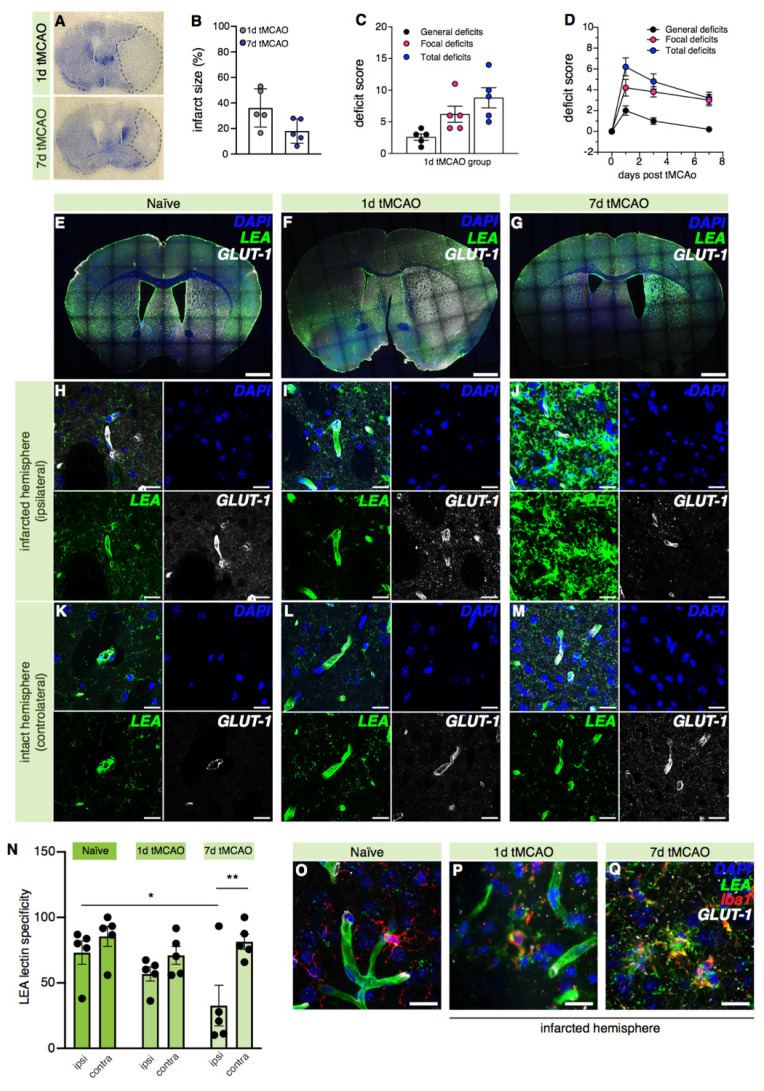
Analysis of the LEA lectin´s labeling of blood vessels in the ischemic core of a post-stroke brain. (**A**) Representative micrograph of the infarcted area (dashed line) in the brain sections of mice 1 and 7 days after transient occlusion of the medial cerebral artery labeled with Nissl staining. Note the infarcted region that appears white. (**B**) Quantification of the infarct size in mice 1 and 7 days post-tMCAo (unpaired *t*-test, *p* = 0.0522); *n* = 5 mice/group. (**C**) Neuroscore values in mice 1 day post-tMCAo; *n* = 5 mice. (**D**) Neuroscore in mice 7 days post-tMCAo; *n* = 5 mice. (**E**–**M**) Representative confocal images of brain sections at 10× magnification (**E**–**G**; scale bars: 1 mm) and related blood vessels at 20× magnification (**H**–**M**; scale bars: 20 µm) of the naïve, 1d tMCAo, and 7d tMCAo mice labeled with the LEA lectin and the GLUT-1 antibody (counterstain: DAPI). (**N**) Quantification of the LEA lectin specificity (two-way ANOVA test, hemisphere effect, *p* = 0.0025; Tukey’s multiple comparisons test for the infarcted hemisphere: naïve vs. 1d tMCAo, *p* = 0.4239; naïve vs. 7d tMCAo, *p* = 0.0112; 1d tMCAo vs. 7d tMCAo, *p* = 0.1619; for the intact hemisphere: naïve vs. 1d tMCAo, *p* = 0.5030; naïve vs. 7d tMCAo, *p* = 0.9451; 1d tMCAo vs. 7d tMCAo, *p* = 0.6983; Sidak’s multiple comparisons test, infarcted vs. intact hemispheres: naïve, *p* = 0.6566; 1d tMCAo, *p* = 0.5601; 7d tMCAo, *p* = 0.0034); *n* = 5 mice/group. * = *p* < 0.05; ** = *p* < 0.01. (**O**–**Q**) Representative confocal images of the LEA lectin and Iba1 signal overlapping in the infarcted hemisphere of the 1d tMCAo and 7d tMCAo mice compared to the naïve mice. Scale bars: 20 µm.

Lastly, we tested whether lectin-based blood vessel labeling may be affected by disease in a mouse model of ischemic stroke where transient occlusion of the middle cerebral artery causes the formation of a necrotic core that is surrounded by a penumbra region (ischemic lesion is indicated with a dashed line in Figure 5A).

The latter is the target of treatment efforts as it is rescuable with adequate strategies [41,42]. Regenerative processes in the peri-infarct region include angiogenesis. Since our results presented above suggest sufficient LEA lectin efficacy of histochemically labeling blood vessels in the brain of WT mice, we tested whether a similar efficacy could be achieved to visualize vascularization after transient middle cerebral artery occlusion (tMCAo) in mice.

Brains were harvested 1 and 7 days post-tMCAo and processed for histology. To determine the infarct size, Nissl-stained brain slides (representatives shown in Figure 5A) were quantified. A smaller yet not significantly different lesion size was observed at 7 days post-stroke compared to 1 day post-stroke (1d tMCAo = 36.10 ± 6.705; 7d tMCAo = 18.00 ± 4.259; *p* = 0.0522, Figure 5B). In addition to the lesion size, neurological function was assessed in both experimental groups (i.e., the 1-day cohort and the 7-days cohort). The total deficit score was mainly driven by focal deficits in both groups (Figure 5C–D). Longitudinal assessment of neurological function in the 7-days cohort revealed declining general and total deficit score that did not show a significant difference (Figure 5D).

Next, the LEA lectin labeling of blood vessels in the infarct core 1 and 7 days post-tMCAo was analyzed and compared with the GLUT-1-immunolabeled blood vessels (Figure 5E–M). Our results showed that 98.78 ± 1.62% of the total GLUT-1-labeled blood vessels were also labeled with the LEA lectin, suggesting that sensitivity of the LEA lectin to blood vessels was unaltered by ischemic brain injury. However, we also observed a significant 59.36 ± 43.12% reduction of the LEA lectin’s specificity towards blood vessels in the infarcted hemisphere 7 days post-tMCAo compared to the naïve condition (Figure 5N, naïve, averaged hemispheres = 73.10 ± 5.928; 7d tMCAo = 32.68 ± 15.51). A 29.30 ± 15.04% reduction of the LEA lectin’s specificity 1 day post-tMCAo was also detected, although not significant (naïve, averaged hemispheres = 73.10 ± 5.928; 1d tMCAo = 56.86 ± 5.410). No significant difference between the control (intact) hemispheres and tissues from the naïve mice was observed (naïve, averaged hemispheres = 73.10 ± 5.928; 1d tMCAo = 71.04 ± 6.8141; 7d tMCAo = 81.43 ± 5.786). The same analysis conducted in the penumbra (Figure S2A–C), showed that the LEA lectin’s specificity is also affected in the peri-infarct region, showing a significant reduction of 64.20 ± 9.89% and 68.94 ± 18.28% for 1 and 7 days post-tMCAo, respectively (Figure S2D; left/infarcted hemisphere: naïve = 84.76 ± 5.031; 1d tMCAo = 30.93 ± 3.821; 7d tMCAo = 26.83 ± 7.060).

In order to investigate the decreased LEA lectin’s specificity in the lesioned hemisphere, we performed counterstaining for the microglia marker ionized calcium-binding adaptor molecule-1 (Iba-1) and the astrocytic marker glial fibrillary acidic protein (GFAP) as both cell types undergo massive activation during brain ischemia [43,44]. Our immunostainings showed apparent co-labeling of the LEA lectin and Iba-1 in the infarcted hemisphere 1 and 7 days post-tMCAo which was absent in the naïve brains (Figure 5O–Q). Interestingly, we did not detect any co-labeling of the LEA lectin with the GFAP astrocytic marker despite apparent astrocytic activation (Figure S2E–G). Thus, our results suggest that the LEA lectin recognizes blood vessels post-stroke; however, it robustly labels other cells in the brain other than endothelial cells, which hampers its suitability as a reliable vessel marker post-stroke.

## 3. Discussion

Lectins have been widely used to label blood vessels in experimental animal models [27,28]. However, whilst a systematic comparison of the labeling efficacies of different lectins have been carried out in organs of invertebrate animal models, e.g., in the heart compartments of zebrafish (*Danio rerio*) and giant danio (*Devario aequipinnatus*) [45], such a comparison has not been carried out in mouse models. This study provides a comprehensive analysis of the labeling efficacy of the IB4, WGA, and LEA lectins in histological staining of blood vessels in brain sections and a comparison of this method with the intracardiac perfusion of the WGA lectin in labeling blood vessels in the brain and other organs. We found that among the three lectins used in this study, the LEA lectin had by far the highest efficacy in labeling blood vessels in free-floating brain sections when using histological approaches.

Our findings also underline the importance of establishing a reliable and consistent method to implement lectin use in histological staining of blood vessels. Indeed, several recently published studies relied on the IB4 lectin to stain blood vessels in embryos [46], in the retina [47,48,49], and also in the brain [50,51]; however, our systematic comparison showed that the LEA lectin is preferable to IB4 in such applications, particularly in the brain of adult mice.

Our study also showed that the lectin with the lowest specificity, the WGA lectin, exhibited a concentration-dependent efficacy in labeling blood vessels in the brain when intracardially injected; furthermore, the highest concentrations used in this study (50 µg/mL and 125 µg/mL) showed similar results to the LEA lectin histological staining in labeling blood vessels in the brain. The evidence that WGA efficacy in labeling blood vessels varies depending on the labeling method (histology on free-floating sections vs. direct labeling of the vasculature during terminal transcardial perfusion) points out that the labeling efficacy of the LEA and IB4 lectins may improve upon direct injection in the bloodstream. However, a previous study where perfusion with the LEA lectin was found to be superior to IB4 to label choroidal and retinal vasculature [52] suggests that an improved method for lectin delivery may not alter the intrinsic difference of the two lectins in labeling blood vessels that we reported in our study. An independent experiment confirmed that the labeling efficacy of intracardial perfusion with the WGA lectin at a 50 µg/mL concentration and the LEA lectin histological staining in labeling blood vessels in most of the other organs analyzed (dura, spinal cord, heart, kidney, muscle) is similar. The liver where the LEA lectin histological staining showed a better signal-to-noise ratio than the intracardially perfused WGA lectin represented the only exception. This result can be explained by the unique structure of blood vessels in the liver. Indeed, the liver sinusoidal endothelial cells are characterized by the presence of open pores, or “fenestrae”, which enable them to control, based on their size, the molecules that can leave the sinusoidal lumen and reach the parenchymal cells [34]. Interestingly, it has been shown that several lectins, including LEA, are able to bind to the sinusoidal endothelial cells in the liver after intravitreal injection; in particular, it appears that sinusoidal endothelial cells in the liver take up lectins in vesicles that resemble, in some cases, coated vesicles of receptor-mediated endocytosis. However, this capability appears to happen only in vivo as it was completely abolished when the lectin was injected after fixation [53]. This finding points out the need to carefully choose the method used for blood vessel staining taking into account the structural differences that different tissues might have.

Since lectins are able to specifically recognize and bind to different carbohydrates through their carbohydrate-binding domains (see Table 1 modified from [30]; nominal carbohydrate specificity was described as in [54]), they have also been used to identify the carbohydrates on the cell surface that are important in cell–cell interactions and during development.

Indeed, Wearne et al. [55] used staining with 34 labeling lectins (using WGA, LEA and IB4, among the others) to establish the different carbohydrate composition of undifferentiated and differentiated human embryonic stem cells (hESCs). Furthermore, they demonstrated a lack of correlation between the specificity of the binding and the location of the staining since lectins with the same carbohydrate specificity would label hESCs differently. This observation could explain the different labeling efficacy of the lectins that we reported in this study. In our study, the WGA lectin showed a diffuse and unspecific labeling of vascular walls and the parenchyma when applied for histological labeling of brain sections; thus, our observations are in line with those of Kostrominova [56] who used the WGA lectin staining to visualize the general background in structures such as the skeletal muscle, the bone, and the ligament/tendon due to its diffuse labeling pattern. However, it should also be noted that WGA has been used since 1978 as a retrograde tracer when injected stereotaxically in the brain [57]; indeed, synaptic plasma membranes are rich in glycoproteins that are bound by WGA, among other lectins [58], and this could explain why the WGA lectin, when applied directly to the brain tissue, showed diffuse staining in our study. 

The carbohydrate composition on cell membranes can change in response to an injury, infection, or pathological state, and lectin-binding patterns may change accordingly. For example, Melo-Junior et al. [59] reported that the binding of concanavalin A (Con A) and the WGA lectin to the human gastric epithelium is altered in patients affected by *Helicobacter pylori* infection; Cuzzocrea et al. [60] showed that the expression of intercellular adhesion molecule 1 (ICAM-1), a glycoprotein expressed by endothelial cells, is increased one hour after reperfusion in a mouse model of ischemia following splanchnic artery occlusion shock (SAO). Such changes have also been reported in hepatocarcinoma (HCC) patients where the decreased expression of C-type lectin domain family 3 member B (CLEC3B) also shows a diagnostic value [61].

In an attempt to address whether pathological conditions might induce modifications of the carbohydrate residues on endothelial cells that could lead to a different lectin binding pattern of blood vessels in the brain, we analyzed the LEA lectin labeling of blood vessels in the brain of the mice that underwent tMCAo to induce stroke. Despite an unaltered sensitivity of the LEA lectin to labeling blood vessels in the ischemic brain, its specificity was significantly decreased in the necrotic core 7 days after tMCAo, as well as in the peri-infarct region at both 1 and 7 days post-stroke. We visually observed the LEA lectin binding to elements in the brain other than blood vessels. By co-immunostaining, we verified an overlapping of the LEA lectin’s signal with the microglial marker Iba1. We did not find co-staining with astrocytic GFAP.

The previous studies reported that lectins are able to label macrophages [31], thus it is not surprising that the brain-resident macrophages, also known as the microglia, can also be labeled by lectins, and indeed *Ricinus communis* agglutinin 1 (RCA-1) has been widely used to stain microglia in rodent [62] and human tissues [63]. Interestingly, a study aiming to prove the beneficial effect of the umbilical cord matrix stem cells transplantation on the inflammatory response after global ischemia in a mouse model of cardiac arrest showed that RCA-1 fails to label microglia when the cardiac arrest does not occur [64], suggesting that RCA-1 may label microglia only in the event of a strong inflammatory response. 

In the initial stages of ischemic stroke, microglia are known to polarize towards the M2 neuroprotective phenotype and switch later on towards the M1 neurotoxic phenotype, especially in the peri-infarct region [65]; this process induces microglia to upregulate a different set of molecules that could cause the LEA lectin to recognize microglia in the event of a strong inflammatory response, similarly to what happens to RCA-1 in the cardiac arrest rat model [64]. Furthermore, it has been reported that microglia-released cytokines interleukin 1α (IL-1α), tumor necrosis factor α (TNFα), and the complement component subunit 1q (C1q) are able to induce polarization of astrocytes towards the neurotoxic A1 phenotype both in vitro and in vivo [66]. As a response to those cytokines, the peri-infarct region undergoes reactive astrogliosis and forms the so-called glial scar, which limits the expansion of the necrotic area [67]. Despite the fact that activated astrocytes are known to adopt a hypertrophic morphology and upregulate GFAP and other molecules [68], they do not appear to express molecules that can be recognized by the LEA lectin as no overlap with the GFAP marker was observed in our study.

The fact that most of our studies were conducted with lectins conjugated to a 488 or FITC dye may raise concerns regarding autofluorescence phenomena. Indeed, autofluorescence of the brain tissue has already been used to assess tridimensional brain morphology and structural organization with high resolution through optical projection tomography [69]. To address whether autofluorescence could play a role in our studies, altering the LEA’s signal-to-noise ratio, we tested this hypothesis using as the negative control the brain tissue that was not stained with lectins, confirming that the signal associated with the LEA lectin staining was indeed specific and not altered by autofluorescence.

Finally, lectins might present some limitations when applied for in vivo labeling of the vasculature; indeed, lectins are known to cause coagulation of red blood cells with potentially fatal consequences. Therefore, lectins are likely limited to sectioned tissues or in vivo delivery shortly before sacrificing the animal, while fluorescent dyes without specific binding capabilities are suitable for imaging over extended periods of time [17,18].

In light of the topics discussed above, we want to encourage the use of the LEA lectin as a reliable marker for labeling blood vessels in immunohistochemical approaches. If the experimental design allows it, direct injection of the WGA lectin at the medium concentration (50 µg/mL) in the bloodstream during terminal intracardial perfusion provides results that are comparable to histochemical techniques, whilst higher doses (125 µg/mL) are able to produce an even better signal-to-noise ratio. Indeed, lectins show specificity towards blood vessels, as suggested by overlapping signals with GLUT-1 staining and Tie2–eGFP reporter mice. However, it has to be noted that the physiological characteristics of the tissue of interest can interfere with the binding of lectins to the carbohydrate residues on cellular surfaces such as in stroke mouse models, therefore limiting their applicability in some experimental settings.

## 4. Materials and Methods

### 4.1. Animals

Adult male C57BL/6 mice (12-week-old) were purchased from Janvier Laboratories and Charles Rivers (for the tMCAo studies) and were used in this study. The mice were kept in the standard laboratory conditions, with a 12 h dark–light cycle and ad libitum access to water and food. The experiments were conducted according to ethics permit Nos. 5.8.18-08269/2019 and 5.8.18-08160/2021 approved by the Malmö–Lund Ethics Committee on Animal Research and Landesamt für Natur-, Umwelt- und Verbraucherschutz (81-02.04.2019.A214/01).

Tie2–eGFP mice purchased from The Jackson Laboratory (stock No. 003658) were bred in the University of Rochester vivarium and used for experiments in adult age after approval of the University of Rochester Medical Center Committee on Animal Resources.

Prox1-eGFP mice [36], back-crossed on a B6 genetic background, were kindly provided by Dr. Kari Alitalo.

### 4.2. Supplies

All the supplies, including lectins, were purchased from Sigma-Aldrich unless otherwise stated. The primary antibodies were purchased from Merck Millipore (Burlington, MA, USA; GLUT-1, cat. No. MABS132), WAKO (Richmond, VA, USA; Iba1, cat. No. 019-19741), Serotec (Oxford, UK; CD68, cat. No. MCA1957), DAKO (Agilent, Santa Clara, CA, USA; GFAP, cat. No. Z033429), and Abcam (Cambridge, UK; CD31, cat. No. ab28364; α-SMA, cat. No. ab5694). The secondary antibodies were purchased from Invitrogen (Waltham, MA, USA). Cy3-conjugated streptavidin was purchased from Jackson ImmunoResearch (Cambridge, UK).

### 4.3. Transient Middle Cerebral Artery Occlusion (tMCAo)

The left middle cerebral artery (MCA) was occluded under isoflurane anesthesia (70% N_2_O, 30% O_2_). The monofilament (9–10 mm coating length, 0.19 ± 0.01 mm tip diameter; Doccol, Sharon, MA, US) was inserted through microincision in the external carotid artery and further advanced to the internal carotid artery. The body temperature was maintained at 37 ± 0.5 °C with a heating pad. The cerebral blood flow (CBF) was monitored with laser Doppler flowmetry (Moor Instruments, Axminster, Devon, United Kingdom). Reperfusion was initiated after 60 min by removing the filament. The mice were sacrificed 1 (*n* = 5 mice) or 7 (*n* = 5 mice) days after tMCAo.

### 4.4. Neuroscore Determination in Mice after tMCAo

We assessed the neurological dysfunction with a neurological scoring system 1, 3, and 7 days after tMCAo [70]. The score ranges from 0 (no deficits) to 56 (poorest performance in all the items) and it is calculated as the sum of the general and focal deficits, including the following general deficits (scores): hair (0–2), ears (0–2), eyes (0–4), posture (0–4), spontaneous activity (0–4), and epileptic behavior (0–12); and the following focal deficits: body symmetry (0–4), gait (0–4), climbing (0–4), circling behavior (0–4), forelimb symmetry (0–4), compulsory circling (0–4), and whisker response (0–4).

### 4.5. Nissl Staining for Infarct Size Determination in Mice after tMCAo

The mice were intracardially perfused with phosphate-buffered saline (1× PBS) followed by perfusion with 4% paraformaldehyde (4% PFA) for fixation of the tissues 1 or 7 days after tMCAo. Brains were harvested and post-fixed overnight in 4% PFA before they were sectioned with a vibratome (100 µm thickness). Every 300 µm, a 20 µm section was collected to perform Nissl staining for infarct size determination. Briefly, the selected sections were mounted on a slide and allowed to dry before they were washed in dH_2_O to remove residual salts. To stain the sections at 60 °C, 0.1% cresyl violet solution (filtered and preheated; Carl Roth, Karlsruhe, Germany) was used. After washing the sections in dH_2_O to remove the excess stain, they were dehydrated through immersion to the increasing concentrations of ethanol. Finally, the sections were immersed in xylene and covered using Eukitt (Orsatec GmbH, Bobingen, Germany). Infarct size was determined from Nissl-stained brain sections using the ImageJ software (NIH, version 2.0.0-rc-31/1.49v; U. S. National Institutes of Health, Bethesda, MD, US). The areas were integrated to calculate the infarct volume. In order to correct for tissue swelling, the infarct volume was expressed as the percentage of the non-infarcted (contralateral) hemisphere.

### 4.6. Histological Labeling of Coronal Tissue Sections of C57BL/6 Mice with the IB4, LEA, and WGA Lectins

The C57BL/6 mice (*n* = 6) were anesthetized with a ketamine–xylazine mix (ketamine, 100 mg/kg; xylazine, 20 mg/kg) perfused with phosphate-buffered saline (1× PBS) followed by a perfusion with 4% PFA for fixation of the tissues. The brain, skeletal muscles, the heart, the kidney, and the liver were harvested and post-fixed overnight in 4% PFA before they were sliced with a vibratome (100 µm thickness). The vertebral column containing the spinal cord was also harvested and post-fixed overnight in 4% PFA before the spinal cord was dissected and processed like the other harvested tissues. The skull cap containing the dura mater was also collected and post-fixed overnight in 4% PFA, followed by dissection. Solutions of the IB4 (Sigma-Aldrich, Burlington, MA, US; lyophilized, cat. No. L2895), LEA-488 (ThermoFisher Scientific, Waltham, MA, U.S; cat. No. L32470), or LEA-649 (Vector Lab, Burlingame, CA, US; cat. No. DL-1178-1) and WGA-FITC (lyophilized, cat. No. L4895) lectins were prepared at concentrations of 5 µg/mL, 10 µg/mL, and 20 µg/mL and used to histochemically stain coronal sections of the organs and the whole-mount dura mater for one hour before the sections were washed three times with 1× PBS and mounted for imaging.

### 4.7. WGA Lectin Perfusion of C57BL/6 Mice

Lyophilized WGA lectin powder was diluted with 1× PBS in three solutions at the concentrations of 5 µg/mL, 50 µg/mL, and 125 µg/mL, respectively. The C57BL/6 mice were anesthetized with a single intraperitoneal injection of the ketamine–xylazine mix (ketamine, 100 mg/kg; xylazine, 20 mg/kg) and then intracardially perfused with 1× PBS. Immediately after that, 5 mL of the WGA lectin solution were injected during the perfusion and allowed to bind to blood vessels for two minutes before further perfusion with 4% PFA for fixation of the tissues. The organs were harvested and post-fixed overnight in 4% PFA before they were sliced with a vibratome (100 µm thickness) and mounted on glass slides for imaging; *n* = 3 mice for the 5 µg/mL group, *n* = 7 mice for the 50 µg/mL group, *n* = 5 mice for the 125 µg/mL group.

### 4.8. GLUT-1, Iba1, CD68, GFAP, CD31, α-SMA Immunostaining, and Biotin Revelation

Tie2-eGFP reporter mice (*n* = 4) were used in order to assess the specificity of lectin labeling of blood vessels. Briefly, the Tie2–eGFP reporter mice were intravenously injected with biotinylated lectin (125 µg/mL) and intracardially perfused with 1× PBS and 4% PFA thereafter. Brain sections obtained from the Tie2–eGFP reporter mice were histochemically stained with Cy3-conjugated streptavidin and the GLUT-1 antibody. Briefly, a blocking solution (0.5% Triton X-100 and 5% serum) was applied for one hour at room temperature (RT) to minimize unspecific antibody binding. Then, the primary antibody α-GLUT-1 (mouse, concentration 1:250) was allowed to label the tissue overnight at 4 °C under gentle shaking. The day after that, the sections were rinsed three times with 1× PBS before the secondary antibody (Cy5 anti-mouse, 1:500) and Cy3-conjugated streptavidin (1:500) were allowed to label the brain sections for 90 min at RT under gentle shaking. Following three rinses with 1× PBS, 4′,6-diamidin-2-fenilindolo (DAPI, 1:1000) was applied before the sections were mounted.

GLUT-1 immunostaining was also used to assess lectin labeling specificity in the mice after tMCAo. Coronal brain sections of the tMCAo and naïve mice were stained using the same procedure as described above with the exception that no biotin revelation was performed, and instead the sections were incubated for 1 h at RT with a solution of the FITC-labeled LEA lectin (5 µg/mL, concentrated) after the secondary antibody incubation and before the DAPI incubation. Co-labeling with the GFAP (rabbit, concentration 1:500) and Iba1 (rabbit, concentration 1:250) primary antibodies was performed in some samples to immunolabel astrocytes and microglia.

CD31 and α-SMA immunostaining was used to assess lectin co-labeling in coronal sections of the brain, kidney, and spleen of wild-type mice. The abovementioned procedure was used for the staining, with the following exceptions for the CD31 staining: (i) the sections were preincubated in a sodium citrate buffer (pH 6.0) to allow antigen retrieval; (ii) a blocking solution (0.3% Triton X-100, 5% BSA, and 1% serum) was also used for primary antibody incubation.

### 4.9. Image Acquisition and Analysis

Mounted organ sections and the whole-mount dura mater were imaged using a confocal scanning microscope (Nikon A1RHD) at 10× magnification for whole section visualization and 20× Nyquist magnification for detailed imaging of the stained blood vessels. The acquired confocal images were analyzed using the Fiji software (NIH, version 2.0.0-rc-69/1.53c). Specifically, the labeling efficacy of the lectins used for histological staining as well as intracardial perfusion was calculated as the ratio of the averaged peak signal at the vascular walls to the background signal (signal-to-noise ratio) obtained by drawing a cross-section of the vessels using the line plot tool in Fiji [71].

For the analysis of the number of vessels/mm^2^, the vessels were counted manually in each image (three acquisitions/animal); for the analysis of the percentage of the area over the threshold, the Fiji software was used to set a manual threshold for each image (3 acquisitions/animal), and the area covered by the stained vessels over the total area was automatically calculated by the software.

For the co-localization analysis of the LEA lectin and GLUT-1 staining, Tie2-labeled vessels were identified and the presence of lectin and GLUT-1 staining in each Tie2-labeled vessel was assessed as 1 (stained) or 0 (non-stained) (six vessels/animal). The same analysis was used to assess the co-localization of the LEA lectin with CD31 and α-SMA staining, with the exception that in this case blood vessels were identified by CD31 or α-SMA labeling.

The LEA lectin’s specificity in infarcted brains was calculated as the percentage of the difference between the total number of elements labeled by the lectin and the number of blood vessels identified by the positivity to GLUT-1 immunostaining in each image (three acquisitions/animal).

Three-dimensional reconstructions and volumetric projections were generated using the Arivis Vision4D software.

### 4.10. Statistical Analysis

All the statistical testing was performed in GraphPad Prism 9 (GraphPad Software; San Diego, CA, US). The Shapiro–Wilk test was used to test the normal distribution of the data. Two groups were compared using unpaired Student’s *t*-test and Welch’s *t*-test or paired Student’s *t*-test according to the experimental design; the nonparametric Mann–Whitney U test was used when the unpaired data followed non-normal distribution, and the nonparametric Wilcoxon test was used when the paired data followed non-normal distribution. For comparison of more than two groups, one-way ANOVA followed by Tukey’s multiple comparisons post hoc test was used. To compare the differences between the intact hemisphere and the infarcted hemisphere between the naïve and tMCAo mice, a two-way ANOVA followed by Tukey’s and Sidak’s multiple comparisons post hoc tests was performed.

All the values are expressed as the means ± SEM; *n* represents the number of animals; *p* < 0.05 was accepted as statistically significant.

## Figures and Tables

**Table 1:** Table summarizing the characteristics of the lectins used in the study; modified from the study by Jilani et al., 2003 [30]; nominal carbohydrate specificity as in the study by Goldstein and Poretz, 2012 [54].

Lectin Abbreviation	Source	Carbohydrate Group	Nominal Carbohydrate Specificity
WGA	*Triticum vulgaris*	N-acetylglucosammine	GlcNAc(β1,4GlcNAc)1-2 > βGlcNAc > Neu5Ac
LEA	*Lycopersicon esculentum* (Tomato)	N-acetylglucosammine	GlcNAcβ1,4GlcNAcβ1,4GlcNAcβ1,4GlcNAc > GlcNAcβ1,4GlcNAcβ1,4GlcNAc > GlcNAcβ1,4GlcNAc
IB4	*Griffonia (Bandeiraea) simplicifolia*	N-acetylglucosammine/ galactose	αGlcNAc = βGlcNAc

## Data Availability

All the data generated or analyzed during this study are included in this published article and the Supplementary Materials.

## Supplementary Materials

The following are available online at https://www.mdpi.com/article/10.3390/ijms222111554/s1, Figure S1: Comparison of the LEA lectin labeling of longitudinally vs. coronally sectioned vessels; Figure S2: Determination of lectin labeling of veins vs. arteries vs. lymphatic vessels; Figure S3: LEA lectin’s specificity in the penumbra of mice after tMCAo; Supplementary Movie 1: Volumetric projection of a lymphatic vessel in the dura of a Prox1–eGFP mouse labeled with the LEA lectin conjugated to AlexaFluor-647.
