# Supplementary material for: Not All Lectins Are Equally Suitable for Labeling Rodent Vasculature
Source: Int J Mol Sci. 2021 Oct 26;22(21):11554. doi: 10.3390/ijms222111554 (PMC8584019; doi:10.3390/ijms222111554)
Supplement: Supplementary file 1 [file ijms-22-11554-s001.zip › ijms-1383156_Supplementary Informations_Oct 26th.pdf]

Supplementary information

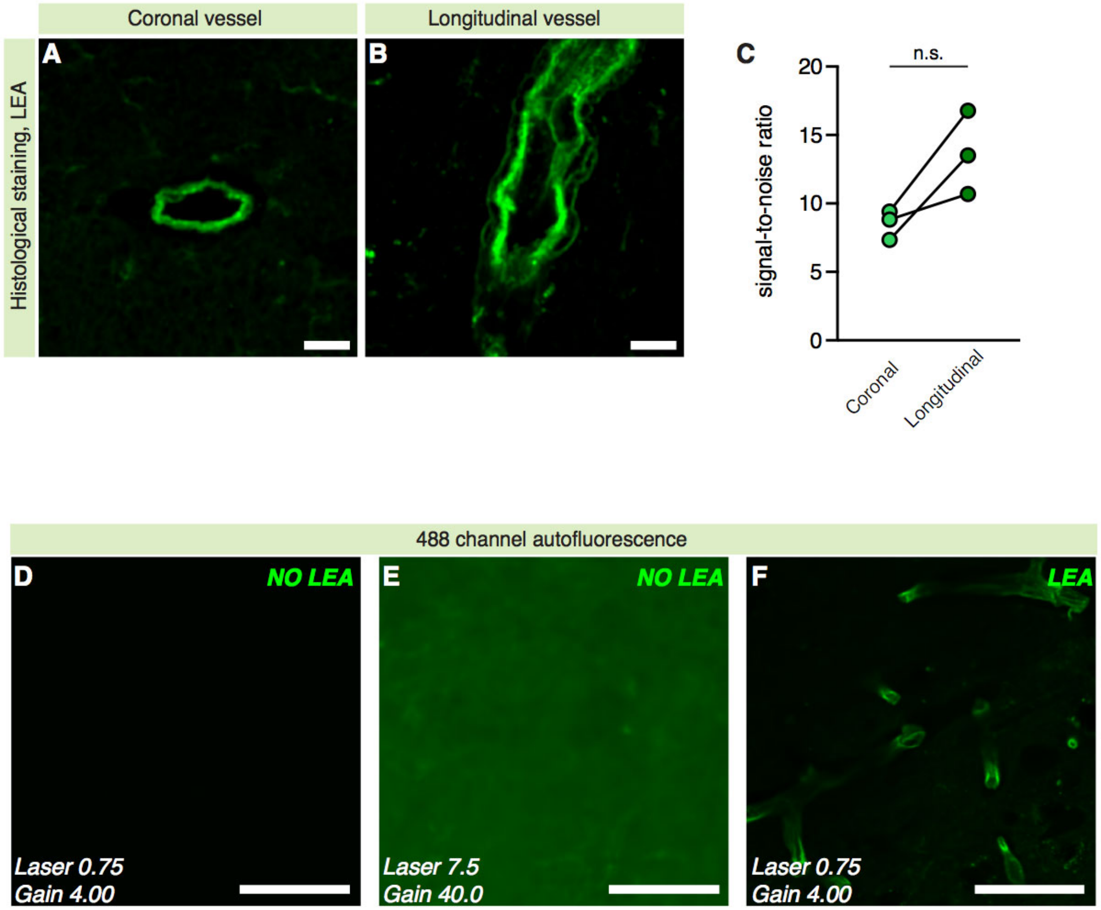

Figure S1: Comparison of LEA lectin labelling of longitudinal vs. coronal sectioned vessels. (A-B) Representative 20× confocal images (scale bars: 10 µm) of blood vessels in the hippocampus (cross-sectional) or in the cortex (longitudinal) labelled with LEA lectin. **(C)** Quantification of the signal-to-noise ratio values (Paired t-test: $p = 0.0917$). n = 3 mice. **(D-F)** Representative confocal images of mouse brain sections non-labelled with LEA lectin, compared to a labelled one to address whether autofluorescence could have an impact on LEA signal. Scale bars: 50 µm.

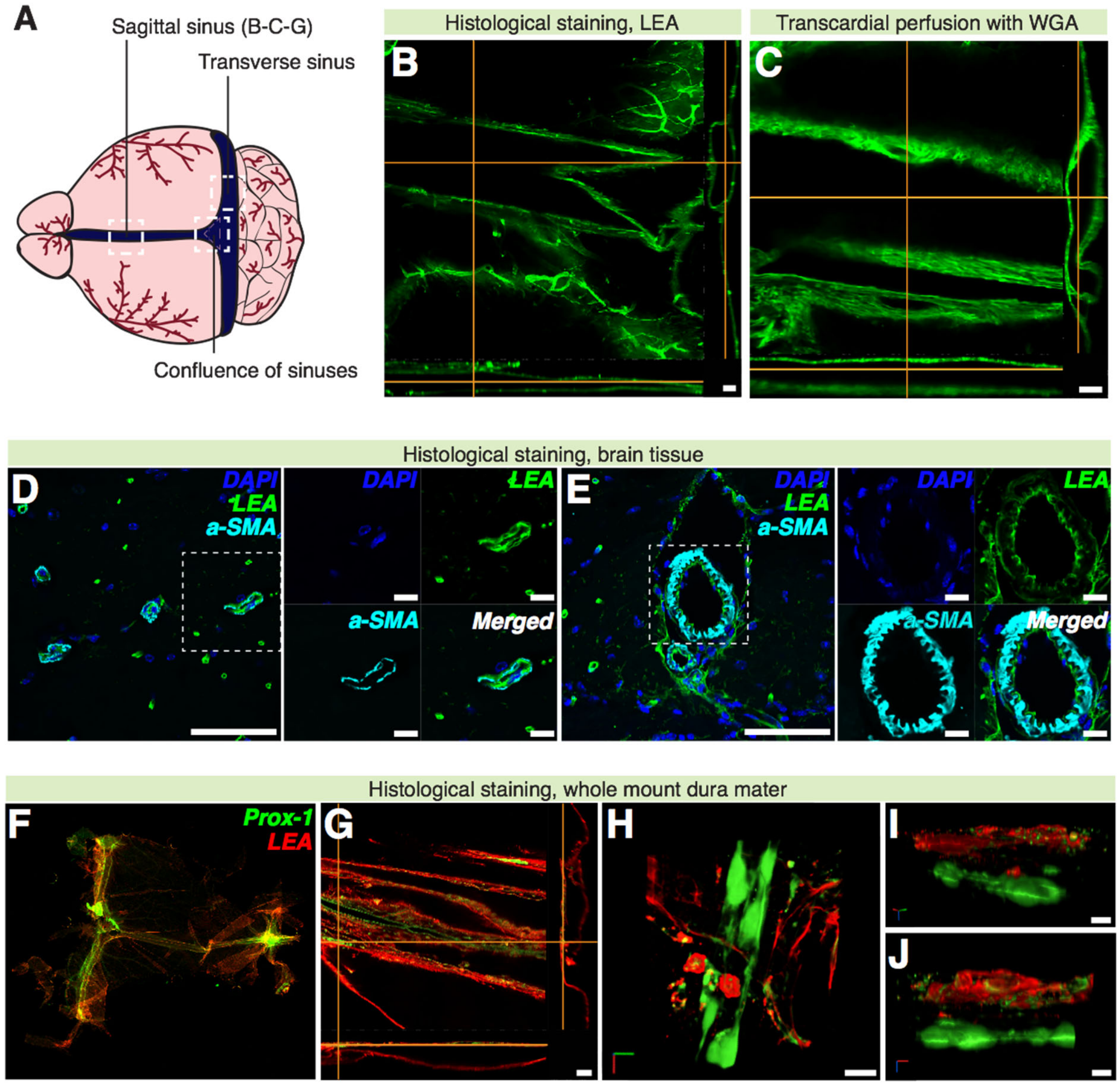

Figure S2: Determination of lectin labelling of veins vs arteries vs. lymphatic vessels. (A) Schematic depicting the sagittal and transverse sinuses in the dura mater. **(B-C)** Representative confocal images of a 3D reconstruction of the sagittal sinus of the dura mater, labelled with LEA lectin histological staining and WGA lectin intracardially perfused, respectively. Scale bar: 50 µm. **(D-E)** Representative confocal images of brain arteries labelled with α-SMA that shows co-labelling with LEA lectin. Scale bar: 50 µm. White dashed squares mark the magnified insets on the right. Scale bars: 10 µm. The images are representative of the co-localization in 5 different mice. **(F)** Representative image of the whole mount dura of a Prox1-eGFP transgenic mice labelled with LEA lectin and **(G)** 3D reconstruction of the sagittal sinus of the dura mater and lymphatic vessels along it. Scale bar: 50 µm. **(H-J)** Volumetric projections of the lymphatic vessel in (G) that shows no overlapping with LEA labelling. Scale bars: 10 µm. The images are representative of 3 different mice.

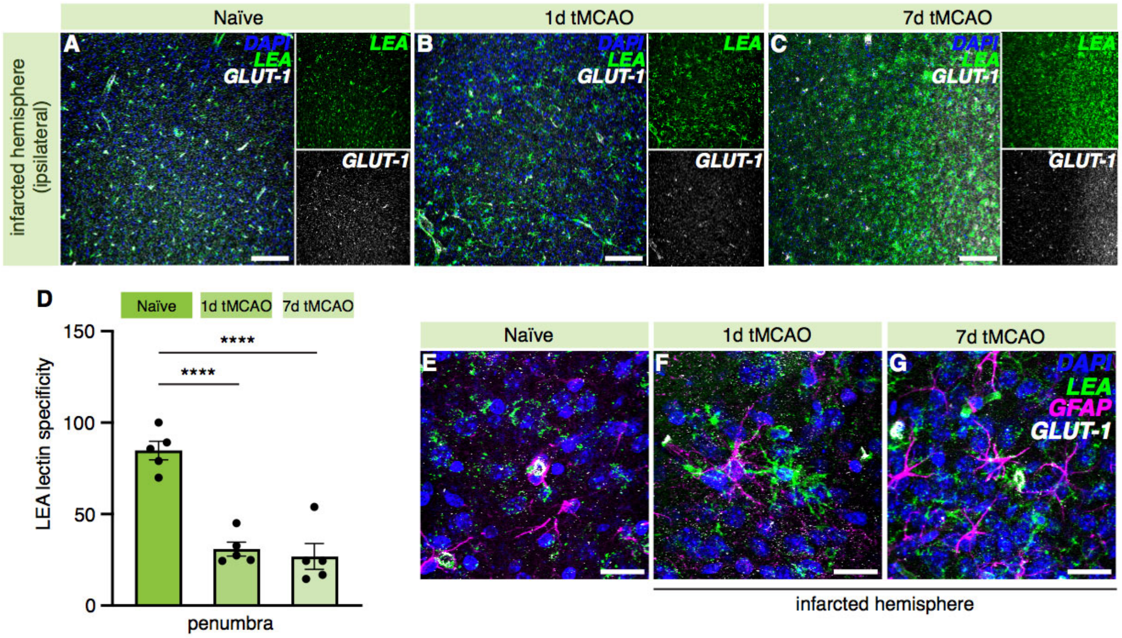

Figure S3: LEA lectin specificity in the penumbra of mice after tMCAo. (A-C) Representative confocal images of blood vessels in the peri-infarct region in the brain of 1d tMCAo and 7d tMCAo mice, labelled with LEA lectin and GLUT-1 antibody and compared to naïve intact brain. Scale bars: 100 µm. **(D)** Quantification of the LEA lectin specificity. (One-Way ANOVA test, $p < 0.0001$; Tukey´s multiple comparisons test for the infarcted hemisphere: naïve vs 1d tMCAo $p < 0.0001$; naïve vs 7d tMCAo $p < 0.0001$; 1d tMCAo vs 7d tMCAo P = 0.8583). n = 5 mice/group. **** = $p < 0.0001$ **(E-F)** Representative 20× confocal images of naïve, 1d tMCAo and 7d tMCAo mice brain sections incubated with LEA lectin, GLUT-1 antibody and GFAP antibody to stain astrocytes. Note that LEA lectin and GFAP signal do not overlap. Scale bars: 20 µm.
